# miRNA and circRNA expression patterns in mouse brain during toxoplasmosis development

**DOI:** 10.1186/s12864-020-6464-9

**Published:** 2020-01-14

**Authors:** Chun-Xue Zhou, Kang Ai, Cui-Qin Huang, Jing-Jing Guo, Hua Cong, Shen-Yi He, Xing-Quan Zhu

**Affiliations:** 1 Department of Pathogenic biology, Shandong University School of Basic Medicine, Jinan, Shandong 250012 People’s Republic of China; 2 State Key Laboratory of Veterinary Etiological Biology, Key Laboratory of Veterinary Parasitology of Gansu Province, Lanzhou Veterinary Research Institute, Chinese Academy of Agricultural Sciences, Lanzhou, Gansu 730046 People’s Republic of China; 3 College of Life Sciences and Fujian Provincial Key Laboratory for the Prevention and Control of Animal Infectious Diseases and Biotechnology, Longyan University, Longyan, Fujian 364012 People’s Republic of China

**Keywords:** *Toxoplasma gondii*, Brain, miRNA, circRNAs, Interaction

## Abstract

**Background:**

Increasing evidence has shown that circular RNAs (circRNAs) are involved in neurodegenerative disorders, but their roles in neurological toxoplasmosis are yet to know. This study examined miRNA and circRNA expressions in mouse brain following oral infection with *T. gondii* Pru strain.

**Results:**

Total RNA extracted from acutely infected (11 days post infection (DPI)), chronically infected (35 DPI) and uninfected mouse brain samples were subjected to genome-wide small RNA sequencing. In the acutely infected mice, 9 circRNAs and 20 miRNAs were upregulated, whereas 67 circRNAs and 28 miRNAs were downregulated. In the chronically infected mice, 2 circRNAs and 42 miRNAs were upregulated, whereas 1 circRNA and 29 miRNAs were downregulated. Gene ontology analysis predicted that the host genes that produced the dysregulated circRNAs in the acutely infected brain were primarily involved in response to stimulus and ion binding activities. Furthermore, predictive interaction networks of circRNA-miRNA and miRNA-mRNA were constructed based on genome-wide transcriptome sequencing and computational analyses, which might suggest the putative functions of miRNAs and circRNAs as a large class of post-transcriptional regulators.

**Conclusions:**

These findings will shed light on circRNA-miRNA interactions during the pathogenesis of toxoplasmosis, and they will lay solid foundation for studying the potential regulation roles of miRNAs and circRNAs in *T. gondii* induced pathogenesis.

## Background

*Toxoplasma gondii,* the causative agent of toxoplasmosis, is a medically important parasite infecting approximately 30% of the world’s population [1]. Immune-potent individuals usually do not show any clinical symptoms, and infection is latent and maintained inside the host as tissue cysts. When infected persons become immunocompromised, such as people suffering from AIDS or organ transplantation, parasite cyst reactivation can lead to acute toxoplasmosis, which includes eye disease, neurological problems and even death [2]. Additionally, infection of pregnant women can cause congenital infection, with dysplasia, hydrocephaly and chorioretinitis occurring in the newborns [3].

The fast proliferating *T. gondii* tachyzoite is fully controlled by the host potent immune response and then transforms into a slowly replicating stage (i.e. bradyzoite) enclosed in tissue cysts, and remains dormant within the central nervous system (CNS) and muscles [4]. Various kinds of brain cells, including microglia, astrocytes and neurons, can be infected. In the chronically infected mice, the parasite cysts were mainly found in the neurons [5]. In a study of congenital toxoplasmosis, cysts were also found in the neurons [6]. In toxoplasma encephalitis (TE), the rostral basal ganglion is the most commonly affected region, followed by the cerebellum and brain stem [7, 8]. In addition, it has been shown that *T. gondii* infection can affect levels of some neurotransmitters, such as dopamine [9]. Positive correlations between *T. gondii* infections and neuropsychiatric disorders like schizophrenia [10, 11], cryptogenic epilepsy [12] and Parkinson’s disease (PD) [13, 14] were revealed in the clinical and epidemiological investigations. However, the mechanisms underlying *Toxoplasma*-induced neuronal disorder in the brain are poorly known.

MicroRNAs (miRNAs) are endogenous small non-coding RNA molecules which regulate gene expression at the post-transcriptional level, and are now widely recognized as essential regulatory molecules involved in neuronal development and function. miRNAs are abundant in the nervous system and a link between miRNAs and the occurrence and development of neurological diseases, such as cerebral ischemia, stroke and neurodegenerative diseases, is becoming increasingly clear [15]. Notably, miRNAs are also found to be regulators of the host response to *Toxoplasma* infection, as miR-146a and miR-155 are highly induced in chronically infected mice brain, but worthy to mention, compared to miR-146a−/− mice, wild type mice show a more severe TE after challenge with genotype II Pru strain [16]. In addition to using host miRNAs for parasite persistence, *T. gondii* infection also modulates host cell miRNA profiles in a timely manner [17]. Another type of non-coding RNA, circular RNA (circRNA), is formed by exon-scrambling and was once largely neglected due to its rarity and lack of biological functions. With the development of high-throughput sequencing technology, thousands of new circRNAs have been identified in various organisms, from microorganisms to mammals [18]. circRNA is found to antagonize miRNA activity by a sponge-like mechanism and thus regulates gene expression at the post-transcriptional level [19]. Several pieces of evidence have shown that circRNAs are most highly and specifically expressed in neural tissues, and some circRNAs are expressed in a specific spatial and temporal pattern in the brain among species. More studies have shown that circRNAs may act as important regulatory factors in the pathogenesis of neurological diseases, such as epilepsy, PD and Alzheimer’s disease (AD) [20]. However, their roles in the occurrence and development processes of neurological toxoplasmosis remain largely unexplored.

To investigate the potential roles of miRNAs and circRNAs in *T. gondii* infection, we performed a high-throughput sequencing analysis to identify differentially expressed miRNAs and circRNAs in mice after acute and chronic *T. gondii* infections, respectively. Furthermore, differentially expressed miRNAs regulated by differentially expressed circRNAs were predicted, and predictive interaction analysis of miRNA-mRNA was also performed. Thus, the present study presented the first transcriptome-wide circRNA landscape and a predictive miRNA-circRNA network in *T. gondii*-infected mice, which provides a valuable dataset that will help elucidate the mechanisms underlying neurological disorders during *T. gondii* infection.

## Results

### Expression of miRNAs in brains from both *Toxoplasma*-infected and normal mice

**Fig. 1:**
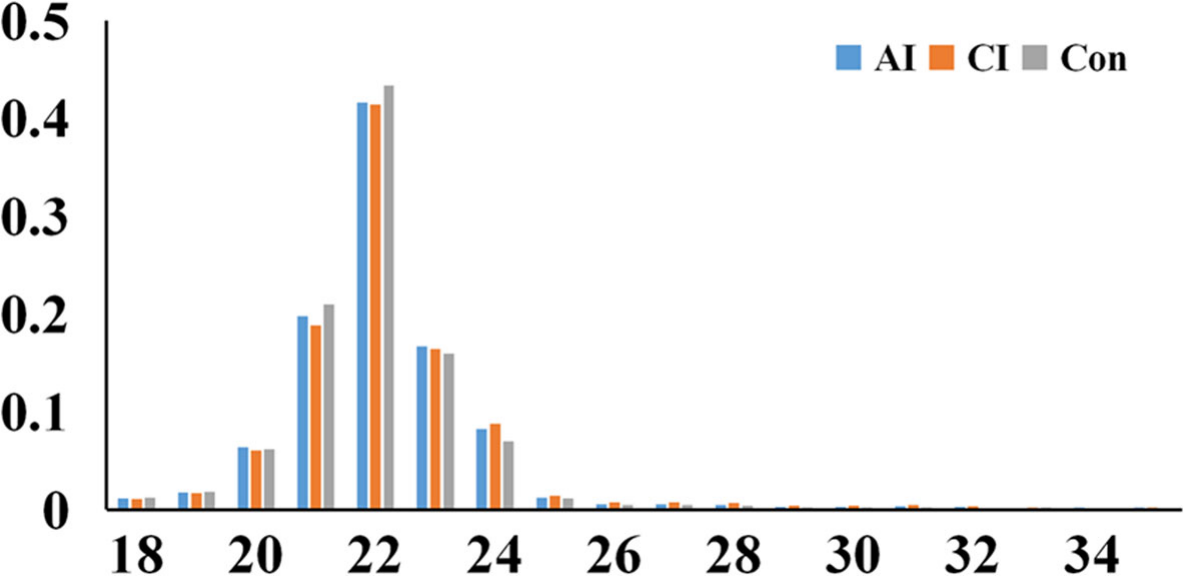
The average length distribution of the sRNA sequences identified in each group. Sample groups including acutely infected, chronically infected and control are labeled as AI, CI, and Con, respectively

Infected mice with *T. gondii* at 11 DPI showed noticeable acute toxoplasmosis signs, including anorexia, hyperpyrexia, and messy hair. All mice survived and restored their physical status at 35 DPI. Initially, we set out to profile the miRNA expression across samples that represent typical infection states. miRNA libraries of brain samples were successfully constructed and summarized in Additional file 1: Table S1. Genome-wide sequencing identified more than 12 million clean reads in each sample. As shown in Fig. 1, the dominant small RNAs (sRNAs) were 20–24 nt in length. More than 93% sRNA sequences were mapped to the reference genome, and the majority of these mapped sRNA sequences were in the forward orientation (Additional file 2: Table S2). Distribution of sRNAs sequences showed that the number of miRNA sequences was higher in the control group (Additional file 3: Table S3). The mapped reads were divided into different categories, and the majority of small RNA reads were belonged to the annotated known miRNAs.

**Fig. 2:**
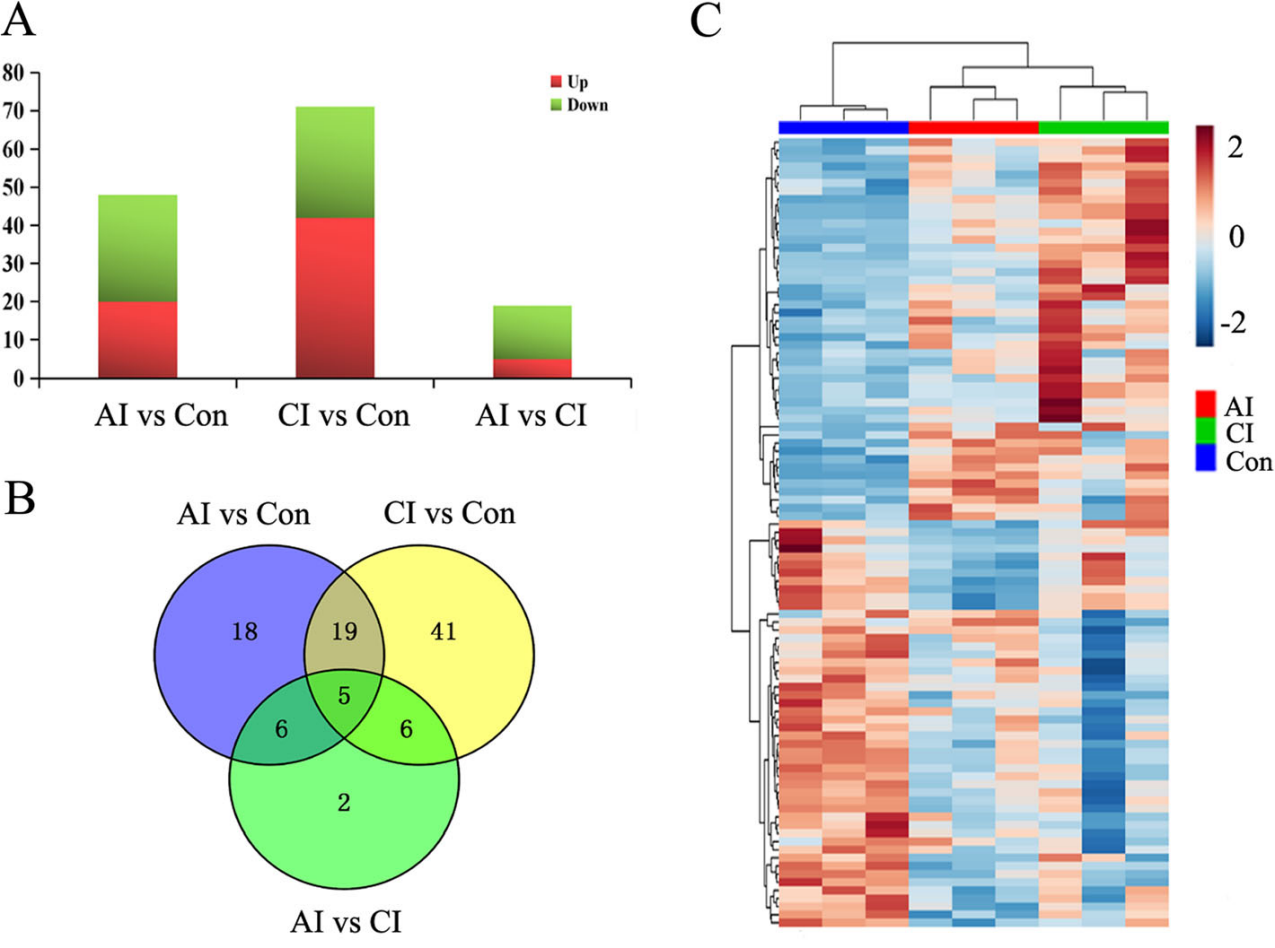
Comparison among normal and parasite infected samples reveals global changes in miRNA expression. **a** Statistics of differentially expressed miRNA among samples. **b** Venn diagram representation of differentially expressed circRNA sharing and exclusive constitutively presented among different time points vs. Control sample. **c** Hierarchical clustering of miRNA expression. miRNA profiles of 9 samples from different groups were clustered. Samples are in columns and miRNA in rows. Sample groups including acutely infected, chronically infected and control, are labeled as AI, CI, and Con, respectively. Brown and blue indicate higher and lower abundance, respectively

We then compared acute infection samples against normal samples and identified 20 over-expressed and 28 under-expressed miRNAs (adjusted *P* value < 0.05) (Fig. 2a). Comparison between chronically infected and control samples revealed more dys-regulated miRNAs, including 42 over-expressed and 29 under-expressed miRNAs. As shown in Fig. 2b, 24 miRNAs were shared between AI vs. Con and CI vs. Con. Worthy to mention, all these 24 miRNAs showed the same variation tendency during parasite infection (Table 1). The clustering of miRNA expression profiles derived from 228 different miRNAs in control and infected samples is shown in Fig. 2c. The tree shows a very good separation between normal and infected samples. However, PCA scores plots did not clearly discriminate chronically infected mice from acutely infected group (Additional file 4: Figure S1A). A recent study showed that infection with *T. gondii* oocysts caused the miRNA perturbation in mice brains [21]. The high-throughput data are available in the NCBI database with the accession number PRJNA418218. As shown in Table 2, the differentially expressed miRNAs shared by both cyst and oocyst infections are listed. It is noteworthy that, mmu-miR-155-5p were up-regulated during the whole infection course in both cyst and oocyst infections, which was further confirmed by the qPCR (Additional file 5: Figure S2).

**Table 1:** The differentially expressed miRNAs shared by both the acute and chronic infections

miRNAs	AI vs. Control		CI vs. Control	
	Log_2_(FC)	Adjusted *p* value	Log_2_ (FC)	Adjusted *p* value
mmu-let-7b-5p	−0.25	1.75E-02	−0.26	2.64E-02
mmu-let-7d-5p	−0.34	1.83E-04	−0.28	1.28E-03
mmu-miR-125a-5p	−0.38	3.17E-02	−0.54	2.12E-02
mmu-miR-142a-3p	2.06	2.85E-24	2.89	4.19E-28
mmu-miR-142a-5p	1.29	1.46E-13	2.34	4.05E-20
mmu-miR-142b	2.06	2.85E-24	2.89	4.19E-28
mmu-miR-146a-5p	1.35	7.93E-24	3.12	8.23E-47
mmu-miR-147-3p	1.47	5.90E-03	1.55	3.15E-03
mmu-miR-155-5p	2.85	8.32E-33	3.42	1.83E-25
mmu-miR-17-5p	0.42	7.05E-03	0.33	4.65E-02
mmu-miR-181a-5p	−0.31	2.25E-03	−0.36	5.40E-05
mmu-miR-203-3p	1.57	2.85E-24	2.10	2.99E-46
mmu-miR-20a-5p	0.54	2.01E-02	0.53	1.05E-02
mmu-miR-20b-5p	1.15	2.22E-02	1.30	5.18E-03
mmu-miR-210-3p	0.73	3.29E-05	0.60	2.90E-02
mmu-miR-219a-2-3p	−0.54	1.67E-04	−0.43	1.57E-02
mmu-miR-21a-3p	1.18	3.74E-02	1.10	4.92E-02
mmu-miR-21a-5p	1.68	1.13E-31	1.55	5.18E-11
mmu-miR-223-3p	2.11	1.32E-17	1.15	1.62E-03
mmu-miR-223-5p	2.28	3.25E-09	1.47	3.50E-03
mmu-miR-3065-3p	−0.48	6.18E-03	−0.70	2.74E-10
mmu-miR-338-5p	−0.48	6.94E-03	−0.69	5.55E-10
mmu-miR-455-3p	−0.78	6.15E-06	−0.72	8.42E-03
mmu-miR-484	−0.61	1.23E-05	−0.39	4.74E-02

**Table 2:** The differentially expressed miRNAs shared by both parasite cyst and oocyst infections

Comparison	miRNA	Cyst infection^a^		Oocyst infection^b^	
		Log_2_(FC)	Adjusted *p* value	Log_2_(FC)	Adjusted *p* value
AI vs. Con	mmu-miR-155-5p	2.85	8.32E-33	1.34	1.31E-03
CI vs. Con	mmu-miR-7043-3p	0.98	6.97E-03	1.08	2.74E-02
	mmu-miR-21a-3p	1.10	4.92E-02	1.23	4.68E-02
	mmu-miR-223-3p	1.15	1.62E-03	1.84	6.98E-08
	mmu-miR-20b-5p	1.30	5.18E-03	1.05	1.49E-02
	mmu-miR-7219-3p	1.41	8.80E-03	2.12	3.56E-05
	mmu-miR-223-5p	1.47	3.50E-03	1.70	8.83E-04
	mmu-miR-21a-5p	1.55	5.18E-11	1.92	3.11E-21
	mmu-miR-147-3p	1.55	3.15E-03	2.91	3.25E-15
	mmu-miR-5107-3p	1.87	1.86E-04	3.00	6.86E-11
	mmu-miR-203-3p	2.10	2.99E-46	2.03	2.87E-23
	mmu-miR-153-5p	2.17	7.22E-07	2.13	5.44E-06
	mmu-miR-142a-5p	2.34	4.05E-20	2.03	4.88E-18
	mmu-miR-142a-3p	2.89	4.19E-28	2.05	2.03E-23
	mmu-miR-142b	2.89	4.19E-28	2.05	2.03E-23
	mmu-miR-146a-5p	3.12	8.23E-47	3.71	9.77E-59
	mmu-miR-155-5p	3.42	1.83E-25	4.04	4.54E-41

^a^indicates results revealed from the present study and ^b^from a previous study performed by Hu et al. (Hu et al., 2018) [21]

### Mapping circRNAs from total RNA-seq data

**Fig. 3:**
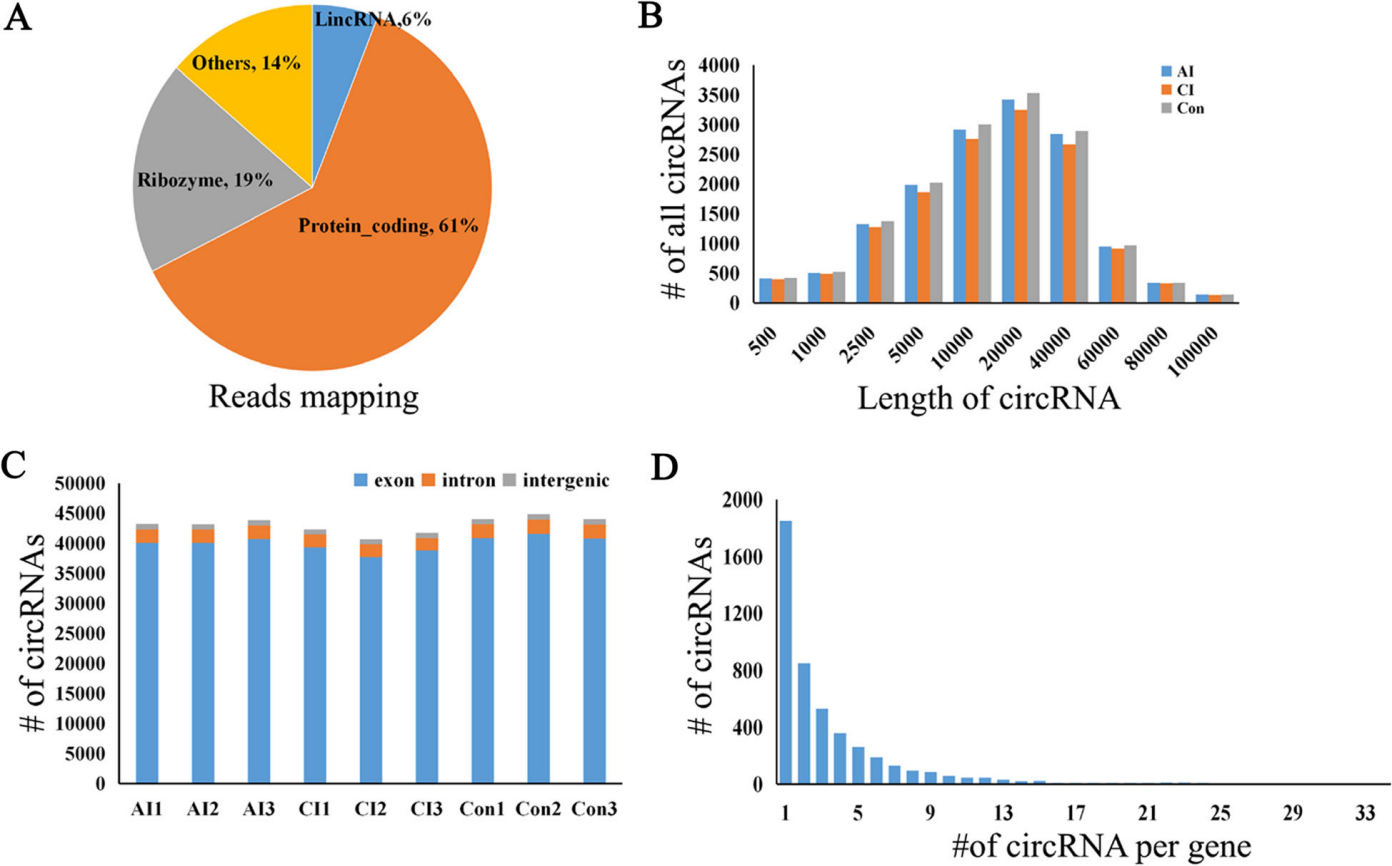
Features of brain circRNAs. **a** Clean reads mapping. **b** The length distribution for identified circRNAs (intergenic circRNAs are excluded). **c** Genome origin of identified circRNAs. **d** Distribution of circRNAs among genes

To investigate whether *T. gondii* infection affects the expression of the host circRNAs, we profiled the brain circRNAs during the toxoplasmosis progression. To obtain sufficient RNA-Seq data, non- polyA-containing RNA, linear RNA and rRNA were removed prior to cDNA library preparation. Tested samples were sequenced on an Illumina HiSeq 4000 with an average data volume of 9 Gb, thereby yielding a total of more than 557 million paired-end reads with a size of 150 bp (Additional file 6: Table S4). Clean reads were mapped to the mouse reference genome (GRCm38/p6) by using Bowtie, and about 61% of these reads consisted of protein coding sequences, whereas smaller fractions aligned with ribozyme and long noncoding RNAs (Fig. 3a). circRNAs were identified and filtered by using both the find_circ and CIRI2 software as previously described [22]. A total of 16,543 circRNAs with a minimum of two reads spanning the back-splicing junction were identified. Annotations for all circRNAs identified in this study are shown in Additional file 7: Table S5. We classified these circRNAs into three groups, namely, exonic circRNAs, intergenic circRNAs and intronic circRNAs. As shown in Fig. 3b, in all of the 9 sample data sets, exonic circRNAs predominated. The size of these circRNA candidates ranged from less than 50 nt to greater than 1500 nt, most of which ranged from 100 nt to 650 nt (Fig. 3c). As shown in Fig. 3d, most genes generated one or two circRNAs, but some genes yielded multiple circRNAs.

### Differentially expressed circRNAs in mice brain during *T. gondii* infection

**Fig. 4:**
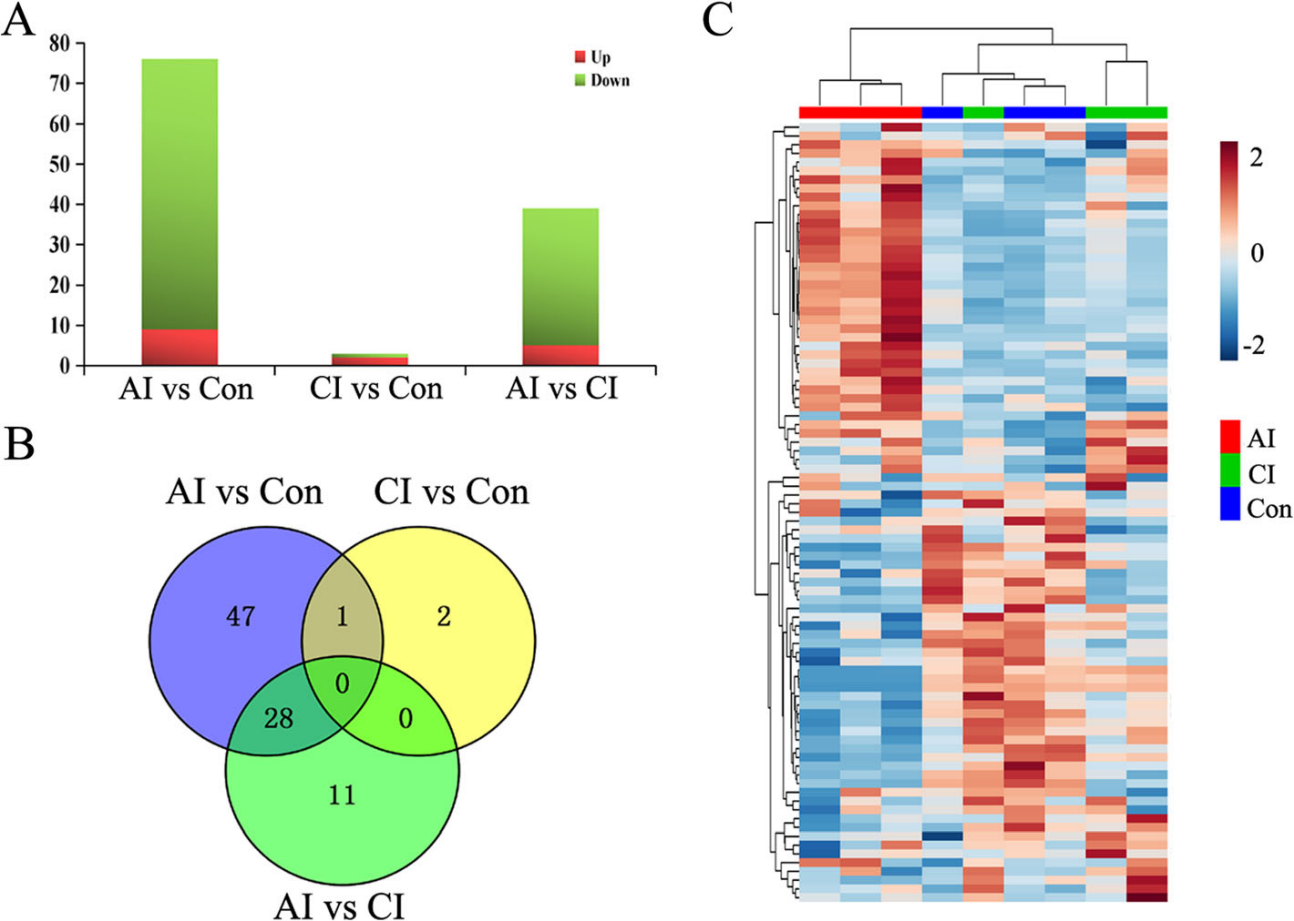
Global view of temporal circRNA expression profiles in mice brain during *T. gondii* infection. **a** Statistics of differentially expressed circRNA among samples. **b** Venn diagram representation of differentially expressed circRNA sharing and exclusive constitutively presented among different time points vs. Control sample. **c** Hierarchical clustering of circRNA expression data. Unsupervised hierarchical clustering was performed using Pearson′s correlation as distance measure and Average-linkage for linkage analysis. Brown and blue indicate higher and lower abundance, respectively. Sample groups including acutely infected, chronically infected and control are labeled as AI, CI, and Con, respectively

We then performed expression profiling to show circRNA variations in mice brain during parasite infection. Expression abundance of circRNAs from nine samples was measured based on TPM, and no abnormal expression was found (data not shown). As a result, compared to control group, 76 circRNAs in acute infection group were detected to be differentially regulated by |log_2_ fold change| ≥0.5, adjusted *P* < 0.05, among which 9 circRNAs were up-regulated while 67 circRNAs were down-regulated. In the chronic infection group, only 3 differentially expressed circRNAs were selected with 2 up-regulated and 1 down-regulated (Fig. 4a). Among these dys-regulated circRNAs, only one was common between AI vs. Con and CI vs. Con, namely novel_circ_0057684 (Fig. 4b). Four circRNAs were selected randomly for validations. As shown in Additional file 8: Figure S3, the results are consistent with predictions. Hierarchical clustering showed that circRNA expression pattern in acute infection group was distinguishable compared to the other two groups (Fig. 4c). Meanwhile, PCA scores plots did not clearly differentiate chronically infected mice from control group (Additional file 4: Figure S1B). These data suggested that the expression of circRNAs in acutely infected brain was severely affected.

### Functional enrichment analysis of differentially expressed circRNAs

**Fig. 5:**
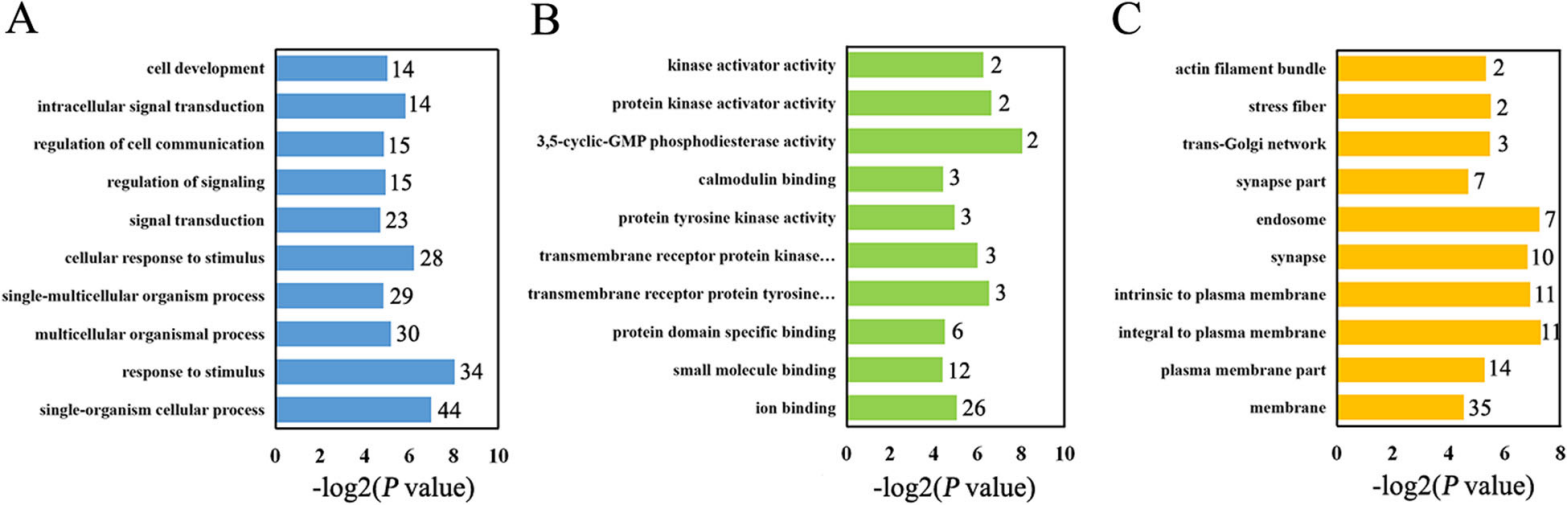
Infection-dysregulated circRNA host gene Gene Ontology (GO) analysis. GO analysis was performed on host genes of significantly dys-regulated (*P* value < 0.05) in the comparison between the acute infection and normal groups. Enriched terms are grouped by GO category-biological process (**a**), molecular function (**b**) and cell component (**c**). The y axis on the left represents the GO term. The number of the input host genes is listed besides the bar. *P* values indicate the significance of enrichment of the input host genes for each GO Term

Comparison of differentially regulated circRNAs and their corresponding host genes between the acutely infected and normal brains are shown in Additional file 9: Table S6. We performed Gene Ontology (GO) analysis on host genes. GO analysis consists of three different aspects, namely biological process, cellular component and molecular function (Fig. 5). Prediction terms with *P*-value less than 0.05 were selected and ranked by enrichment score (−log_2_ (*P*-value)), and the top 10 generally affected GO terms in each categories are listed. The most enriched biological process terms were related to cellular process, signal transduction and response to stimulus, such as “response to stimulus (GO:0050896)” and “single-organism cellular process (GO:0044763)”. The most enriched molecular function terms were mostly about binding activity and kinase activity, such as “ion binding (GO: 0043167)” and “transmembrane receptor protein kinase activity (GO: 0019199)”. As for cell component, the top three enriched terms were “integral to plasma membrane (GO: 0005887)”, “endosome (GO: 0005768)” and “intrinsic to plasma membrane (GO: 0031226)”.

### Analyses of of circRNA-miRNA and miRNA-mRNA interactions

**Fig. 6:**
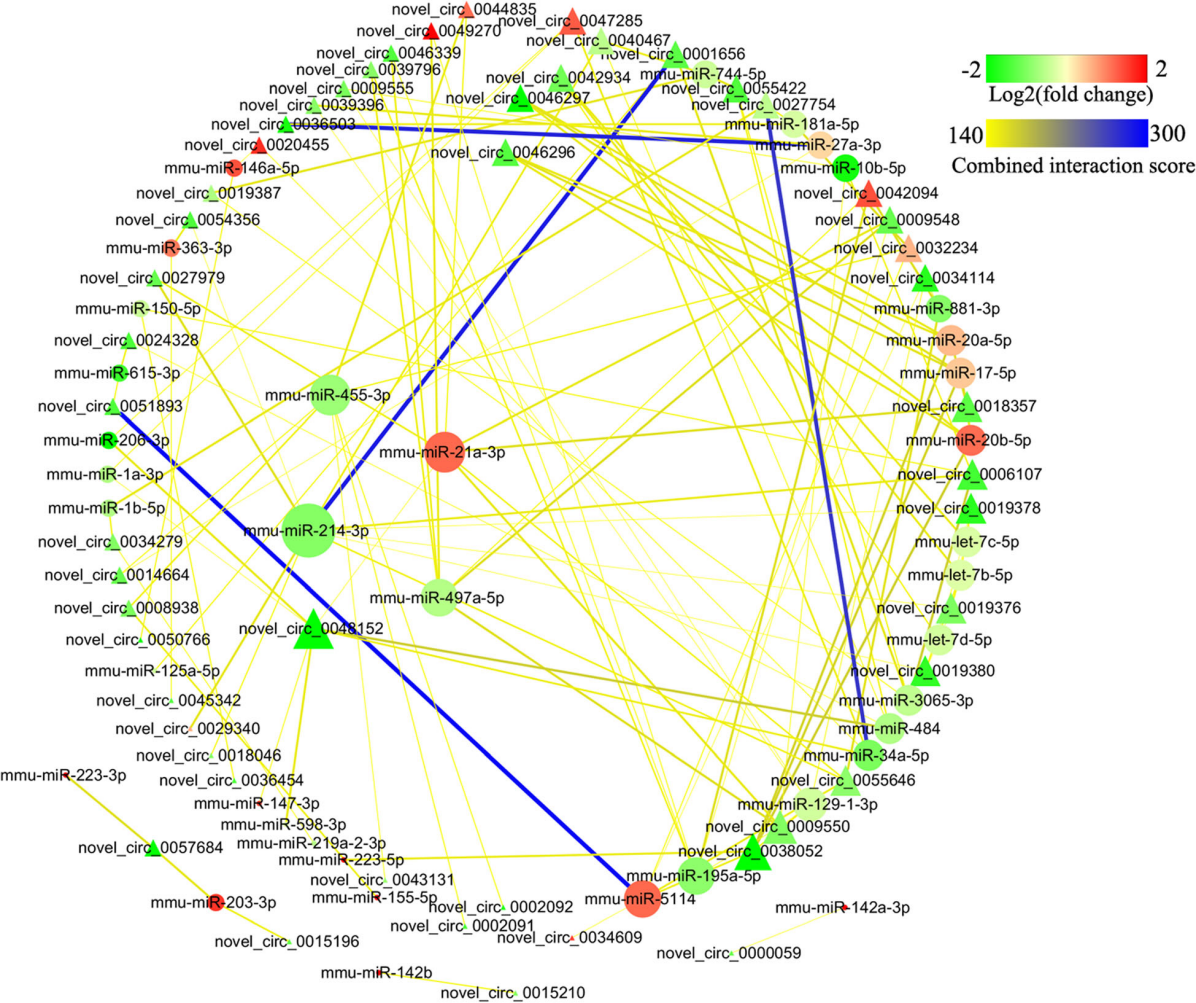
CircRNA-miRNA interaction network. The network consists of 50 circRNAs and 38 miRNAs identified between the acutely infected and normal mouse brains. Triangle indicates circRNA and circle indicates miRNA. The red node indicates upregulated or green node indicates downregulated RNAs (log_2_ fold change). The node size indicates high interaction degree (large) or low degree (small). RNAs that are associated to each other are linked by an edge. The color of the edge indicates miRanda combined interaction score for that particular circRNA-miRNA pair

circRNAs are a unique class of endogenous non-coding RNAs which act as miRNA decoys or sponges to regulate gene expression. Therefore, an integrated analysis of the expression profile from circRNA-miRNA predictive interactions was performed based on the high-throughput RNA sequencing results. First, using miRanda software, the binding site analysis and predictive interaction analysis of circRNAs-miRNAs identified in the comparison between acutely infected and normal brains were performed. As shown in Fig. 6, 38 dys-regulated miRNAs and 50 dys-regulated circRNAs were involved in this network. The data used to create Fig. 6 are listed in Additional file 10: Table S7. Down-regulated mmu-miR-214-3p is the most frequently targeted miRNA by 11 circRNAs, followed by up-regulated mmu-miR-21a-3p, down-regulated mmu-miR-455-3p and mmu-miR-497a-5p. It is worth noting that the novel_circ_0048152 showed the largest interaction network. All the above five RNAs were selected to validate the expression profiles obtained by RNA-Seq, and the qPCR results were consistent with the RNA-Seq findings (Additional file 11: Figure S4). Next, predictive miRNA-mRNA interacting pairs were ranked by the *P* value of the hypergeometric distribution. As shown in Table 3, three relationships between miRNA and targeted mRNA (|fold change|≧1.5, *P* < 0.05) in the acute infection stage and five relationships in chronic infection stage were revealed, respectively. As shown in Additional file 12: Figure S5, the expression levels of these differentially expressed mRNAs were consistent with the high-throughput sequencing data.

**Table 3:** Pairwise co-expression analysis of all profiled miRNAs and mRNAs during the whole *T.gondii* infection

Comparison	miRNA				Target gene			
	Name	Log_2_(FC)	*P* value	Adjusted *P* value	Name	Log_2_(FC)	*P* value	Adjusted *P* value
AI vs. Con	mmu-miR-34a-5p	−1.02	7.21E-14	7.85E-12	SERPING1	4.09	2.93E-02	0.40
	mmu-miR-34a-5p	−1.02	7.21E-14	7.85E-12	TRIM21	4.24	2.33E-02	0.38
	mmu-miR-214-3p	−0.95	1.05E-03	2.51E-02	DGCR6	0.89	4.81E-02	0.45
CI vs. Con	mmu-miR-125a-3p	−0.68	2.63E-03	3.53E-02	DGKZ	−0.58	6.56E-03	0.80
	mmu-miR-484	−0.39	4.33E-03	4.74E-02	DGKZ	−0.58	6.56E-03	0.80
	mmu-miR-673-5p	−0.67	2.61E-03	3.53E-02	IRF1	4.59	4.65E-02	0.81
	mmu-miR-7043-3p	0.98	3.09E-04	6.97E-03	TGFBR1	0.71	4.05E-02	0.81
	mmu-miR-7043-3p	0.98	3.09E-04	6.97E-03	IFI44	5.08	4.72E-03	0.74

## Discussion

A previous study indicated that *T. gondii* infection could modulate the behavior of the intermediate hosts with the presence of tissue cysts in the brain, and the infected rodents showed impaired learning and memory [23]. In humans, increased rates of psychiatric disorders and suicide, and decreased psychomotor performance have been associated with persistent *T. gondii* infection [24, 25]. In order to reveal the mechanisms underlying neuronal disorder, we need to better understand brain gene expression and regulation induced by *T. gondii* infection.

Previous studies have shown that more genes were differentially expressed during chronic infection compared to acute infection after type II strain infection [26, 27]. Among these differentially expressed genes, more genes were up-regulated and were primarily involved in immune regulation and cell activation. The fast development of RNA-seq technology offers unique opportunities to discover novel regulating factors in toxoplasmosis pathogenesis. In this study, we comprehensively profiled the miRNA and circRNA expressions of mice brains in order to further our understanding of the disease pathogenesis. Using RNA-seq data, we revealed a global bias for miRNA and circRNA accumulation in *T. gondii* infected mouse brains.

miRNAs, widely spread in animal cells, can bind to the complementary site on the 3′ untranslated region (UTR) of the targeting mRNAs and thus facilitate mRNA degradation or translation inhibition on a post-transcriptional level. In a previous study, 637 miRNAs were identified in Kunming mouse brains at 14 and 21 days post infection with *T. gondii* cysts, which is a phase of rehabilitation [17]. However, the miRNAs expression has never been profiled in the acute and chronic infection stages after *T. gondii* infection. In this study, we identified 1314 known miRNAs and 91 novel miRNAs, of which 48 and 71 differentially expressed miRNAs were found in acute and chronic infection, respectively. Mmu-miR-155-5p is the most up-regulated miRNA during the whole infection course, which also showed significantly perturbed expression in oocyst-induced toxoplasmosis [21]. mmu-miR-155-5p showed an elevated level in chronic infection compared to acute infection, which might be resulted from the increased high chronic cyst burdens. Several studies provide evidences that miR-155 (the precursor of miR-155-5p) is involved in the innate and acquired immune responses, hematopoiesis and autoimmune disorders [28–30]. More evidences show that elevated levels of miR-155 occur in the formation and development of several tumors, such as leukemias and breast cancer. Both the levels of mmu-miR-142a-3p and mmu-miR-142a-5p were up-regulated in this study. miR-142a-3p has been identified as an essential player in the formation and differentiation of hematopoietic stem cells, in which miR-142a-3p directly targets transcription factor p53 which is involved in the biological process of hematopoietic stem and progenitor cells [31]. Transplantation of mesenchymal stem cells (MSCs) shows good therapeutic effects on acute lung injury, during which Beclin-1 protein is up-regulated. miR-142a-5p is found to negatively regulate Beclin-1 production, and its expression is inhibited during the transplantation of MSCs [32].

Over-expression of the miR-223 significantly reduced *Plasmodium falciparum* infection during the intraerythrocytic life cycle [33]. In this study, both mmu-miR-223-3p and mmu-miR-223-5p were up-regulated, which might be involved in host defense against *T. gondii* infection. Mmu-miR-203-3p was also constantly up-regulated during the whole infection course. Interestingly, the miR-203 functions as a tumor suppressor and is down-regulated in many kinds of cancers, such as primary prostatic tumors, cervical cancer and rhabdomyosarcoma [34–36]. In the present study, mmu-miR-185-3p was the most down-regulated miRNA. More evidences showed that the expression level of miR-185 decreased in carcinogenesis, in which miR-185 suppresses tumor proliferation by directly targeting DNA methyltransferase 1 (DNMT1) [37, 38].

circRNAs are produced by RNA back splicing and highly prevalent in the eukaryotic transcriptome [39]. While the functions of circRNAs remain poorly known, some circRNAs regulate gene expression by acting as miRNA sponges. For instance, a heart-related circRNA (HRCR) was found to function as an endogenous miR-223 sponge, which might be involved in the protection of the heart from pathological hypertrophy and heart failure [40]. In the present study, a large number of circRNAs were identified in both infected and normal mouse brains. Most circRNAs have low abundance, and certain circRNAs are expressed in one gene locus. Interestingly, 76 and 3 differentially expressed circRNAs were identified in the acute and chronic infection stages, respectively, which suggests that these circRNAs may play key roles in toxoplasmosis pathogenesis. In order to explore the roles of these differential circRNAs in mouse toxoplasmosis, GO analysis was used to annotate the biological functions of host linear transcripts in the acute infection stage, and the results showed that a significant amount of GO terms were related with the cellular process and binding activity. circRNA-miRNA predictive interaction networks were also established to predict the relationships between circRNAs and miRNAs. Generally, a circRNA harbors more binding sites for different miRNAs, which means that the circRNA may play multiple functional roles in the regulation of miRNA target gene expression. In this study, it was found that the novel_circ_0048152 showed the largest interaction network and might potentially interact with seven miRNAs including mmu-miR-206-3p, mmu-miR-1a-3p, mmu-miR-598-3p, mmu-miR-147-3p, mmu-miR-484, mmu-miR-34a-5p and mmu-miR-21a-3p. Further biological roles of the novel_circ_0048152 still need to be explored. Moreover, we found that mmu-miR-214-3p, mmu-miR-21a-3p, mmu-miR-455-3p and mmu-miR-497a-5p were potentially co-expressed with multiple circRNAs. However, their functional roles during toxoplasmosis have never been reported.

## Conclusions

The present study revealed unique sets of miRNAs and circRNAs along with their expression profiles. Their potential roles were predicted by bioinformatics softwares. Predictive interaction networks were constructed for both miRNA-mRNA and circRNA-miRNA. Our data lay a solid foundation for studying the functions of miRNAs and circRNAs in toxoplasmosis pathogenesis, which will be performed in further experimental studies.

## Methods

### Mice and parasite

Six to eight-week-old female BALB/c mice were purchased from the Experimental Animal Center of Lanzhou Veterinary Research Institute, Chinese Academy of Agriculture Sciences, PR China. Strict animal handling guidelines were followed in this study. All animals had free access to food and water. They were kept at 22 °C in a 12/12 h light/dark cycle.*T. gondii* type II Pru strain was used in this study, and was maintained throughout the study via oral inoculation of cysts in mice. Cysts were obtained from brain tissues of infected Kunming mice after 60 days post infection (DPI).

### Mouse infection and sample collection

Nine BALB/c mice were randomly assigned to three experimental groups and each group contains three individuals. Six mice were infected orally with ~ 10 freshly prepared *T. gondii* cysts by gavage. Meanwhile, three mock-infected (control) mice received 100 μL saline alone. All infected mice were checked daily for the development of clinical signs of toxoplasmosis. At 11 and 35 DPI with *T. gondii* cysts, infected (*n* = 3 at each time point) and control mice (n = 3) were anesthetized with 5% isoflurane gas at around 10 a.m., and then sacrificed by cervical dislocation. The brain tissues were collected and immediately snap-frozen in liquid nitrogen and kept at − 80 °C until use.

### RNA extraction

Total RNA was extracted from brain tissues using Trizol method (Invitrogen, Carlsbad, CA, USA). The purity of RNA was determined using the NanoPhotometer® spectrophotometer (IMPLEN, CA, USA). Integrity of RNA was confirmed using the RNA Nano 6000 Assay Kit of the Bioanalyzer 2100 system (Agilent Technologies, CA, USA). The concentration of RNA was quantified using Qubit® RNA Assay Kit in Qubit® 2.0 Flurometer (Life Technologies, CA, USA). RNA samples were stored at − 80 °C until used.

### RNA sequencing

The detailed method for mRNA sequencing was described previously [41]. Three micrograms of the total RNA per sample was used for the construction of the small RNA library. Sequencing libraries were constructed using NEBNext® Multiplex Small RNA Library Prep Set for Illumina® (NEB, USA.) according to the manufacturer’s recommendations. The quality of sequencing library was evaluated on the Agilent Bioanalyzer 2100 system using DNA High Sensitivity Chips. Small RNA sequencing libraries were constructed with HiSeq 2500 sequencing platform and 50 bp single-end reads were generated. Raw data of fastq format were firstly processed through in-house perlscripts. Q20, Q30, and GC-content of the clean data were calculated. In order to analyze the small RNA expression and distribution on the genome, small RNA tags were mapped to reference sequence by Bowtie [42]. MiRBase 20.0 was used to align small RNA to the miRNA precursor to obtain the known miRNA. By exploring the secondary structure, the Dicer cleavage site and the minimum free energy of the unannotated small RNA tags, softwares miREvo and mirdeep2 were integrated to predict novel miRNAs [43, 44].

For the construction of circRNA library, 5 μg RNA per sample was used as input material. Ribosomal RNA was removed by Epicentre Ribozero™ rRNA Removal Kit (Epicentre, USA) followed by the linear RNA digestion with RNase R (Epicentre, USA). The sequencing libraries were constructed by NEBNext® Ultra™ Directional RNA Library Prep Kit for Illumina® (NEB, USA) following the manufacturer’s instructions. According to the manufacturer’s instructions, circRNA sequencing libraries were constructed with HiSeq 4000 sequencing platform and 150 bp paired-end reads were generated. Reference mouse genome was downloaded from the NCBI genome website directly. Index of the reference genome was built using bowtie2 v2.2.8 and paired-end clean reads were aligned to the reference genome using Bowtie. The mapped reads were removed. The unmapped reads, including splice junctions-aligned reads were further processed by find_circ and CIRI2 [45, 46]. At last, the circRNAs that contain splice junctions-aligned reads were identified. The intersection between the two algorithms for circRNA prediction was selected.

### RNA quantification and differential expression analysis

The FPKM (fragments per kilo-base of exon per million fragments) of the coding genes was calculated using Cuffdiff (v.2.1.1) to evaluate the mRNA expression level in each sample [47]. The expression levels of miRNAs and circRNAs were estimated by TPM (transcripts per million reads) through the following criteria: Normalized expression = actual miRNA readcount or circRNA junction readcount× 1,000,000/libsize (libsize is the sum of miRNA or circRNA readcount) [48].

Differential expression analysis of two groups was conducted using the DESeq 2(1.14.1). The *P*-values were adjusted using the Benjamini & Hochberg method. Corrected *P*-value of 0.05 was set as the threshold for significantly differential expression by default [49]. By using metaboanalyst 4.0 online software, these differentially expressed RNAs were selected to perform hierarchical cluster analysis and principal component analysis (PCA) [50].

### Quantitative PCR

Total RNA was extracted using Trizol reagent (Life Technologies). To quantify the amount of mature miRNA, we used the miRcute Plus miRNA qPCR Detection Kit (SYBR Green) (FP411, Tiangen Biotech, Beijing, China) and small nuclear U6 as an internal standard. The reactions were performed at 95 °C for 15 min, 94 °C for 20 s, 60 °C for 30 s, 72 °C for 34 s. All reactions were run in triplicate. To quantify the amount of circRNAs, cDNA was synthesized with the HiScript II Q RT SuperMix (Vazyme, Nanjing, China). Quantitative real-time PCR (qRT-PCR) was performed with a ChamQ SYBR qPCR Master Mix kit (Vazyme, Nanjing, China) and GAPDH was used as a normalization control. The reactive cycle consisted of 95 °C for 30 s, then 40 cycles of 95 °C for 5 s and 60 °C for 34 s. A set of divergent primers (the forward primer being located downstream of the reverse primer) were designed and used for backspliced circRNA detection. All PCR primers were designed using oligo7 software and are listed in Additional file 13: Table S8. The qRT-PCR was run on a Bio-Rad CFX connect Real-Time PCR Detection System (BIORAD, USA) and the qRT-PCR data were analyzed using the comparative 2^−ΔΔCT^ method.

### miRNA target gene prediction and functional analysis

The miRanda, PITA and RNAhybrid softwares were used to predict potential target genes of all the differentially expressed miRNAs. An intersection among the three combined predictions were selected. Threshold parameters for PITA were set as follows: max target length: 50,000; energy cutoff: − 10; *p*-value cutoff: 0.05. Parameters for RNAhybrid were set as follows: utr: 3utr.fa; mir: mature.fa. Functional annotation of the predicted microRNA targets were conducted using the GO database. GOseq package was downloaded (http://bioinf.wehi.edu.au/software/goseq/) and used in this study [51]. GOseq based on Wallenius non-central hyper-geometric distribution was implemented for GO enrichment analysis.

### Prediction of circRNA-miRNA interactions

circRNAs function as miRNA sponges and affect miRNA-mediated regulation of target gene expression via natural sequestration or competitive suppression of miRNA activity [52]. circRNA-miRNA interactions were explored with these obtained differentially expressed miRNAs and circRNAs. By using miRanda software, circRNA-miRNA predictive interaction network was built based on the prediction and analysis of miRNA binding sites and the correlations between circRNA and miRNA that were ranked as previously described [53]. In this study, we set the Smith-Waterman hybridization alignments match score higher than 140 and the minimum free energy of the duplex structure less than − 10 to guarantee the reliability of our results. Other setting parameters were as follows: gap open penalty:-9; gap extend penalty: − 4; scaling parameter: 4. Cytoscape 3.71 software was used to diagram the map of the circRNA-miRNA interaction network [54].

### Statistical analysis

Statistical analyses were performed using SPSS 20.0 software. All data were expressed as the mean ± SEM. *P* value less than 0.05 was considered statistically significant. Student’s *t* test was used to compare the qPCR results.

## Supplementary information

**Additional file 1: Table S1.** Reads quality of miRNA libraries.

**Additional file 2: Table S2.** Statistics of small RNA reads mapped to the reference sequence.

**Additional file 3: Table S3.** RNA reads counts in the infected and control brains.

**Additional file 4: Figure S1.** Principal Component Analysis (PCA) score scatter plots of miRNA (A) and circRNA (B) obtained from high-throughput RNA sequencing. Ellipses enclose the 95% confidence intervals estimated by the sample means and covariances of each group.

**Additional file 5: Figure S2.** Validation of the expression of mmu-miR-155-5p using qPCR analysis. Y axis indicates the relative expression with log_2_ fold change.

**Additional file 6: Table S4.** Reads quality of circRNA libraries.

**Additional file 7: Table S5.** Annotation for all circRNAs identified in this study.

**Additional file 8: Figure S3.** Validation of circRNA expression by qPCR. Y axis indicates the relative expression with log_2_ fold change.

**Additional file 9: Table S6.** Differentially expressed circRNAs and their corresponding host genes.

**Additional file 10: Table S7.** The data used to construct Fig. 6.

**Additional file 11: Figure S4.** qPCR verification.

**Additional file 12: Figure S5.** Validation of mRNA expression by qPCR. Y axis indicates the relative expression with log_2_ fold change.

**Additional file 13: Table S8.** Primers for miRNA and circRNA q-PCR analysis.

## Data Availability

The high-throughput data are available in the NCBI database. The bioproject accession numbers for the mRNA-seq, miRNA-seq and circRNA-seq data reported in this paper are PRJNA516994(https://www.ncbi.nlm.nih.gov/bioproject/PRJNA516994), PRJNA516517(https://www.ncbi.nlm.nih.gov/bioproject/?term=PRJNA516517) and PRJNA516718(https://www.ncbi.nlm.nih.gov/bioproject/?term=PRJNA516718), respectively. The full dataset is also available from Chun-Xue Zhou upon request (zhouchunxue23@163.com).

## Abbreviations

AI: Acute infection
AIDS: Acquired immune deficiency syndrome
CI: Chronic infection
DGCR6: DiGeorge syndrome critical region gene 6
DGKZ: Diacylglycerol kinase zeta
DNMT1: DNA methyltransferase 1
DPI: Days post infection
FC: Fold change
FPKM: Fragments per kilo-base of exon per million fragments
GAPDH: Glyceraldehyde-3-phosphate dehydrogenase
GO: Gene Ontology
IFI44: Interferon-induced protein 44 like
IRF1: Interferon regulatory factor 1;MSCs Mesenchymal stem cells
nt: Nucleotide
SERPING1: Serine (or cysteine) peptidase inhibitor, clade G, member 1
SPF: Specific-pathogen-free
TGFBR1: Transforming growth factor, beta receptor I
TRIM21: Tripartite motif-containing 21
