# Supplementary material for: miRNA and circRNA expression patterns in mouse brain during toxoplasmosis development
Source: BMC Genomics. 2020 Jan 14;21:46. doi: 10.1186/s12864-020-6464-9 (PMC6958735; doi:10.1186/s12864-020-6464-9)
Supplement: Supplementary file 1 — Additional file 1: Table S1. Reads quality of miRNA libraries. [file 12864_2020_6464_MOESM1_ESM.doc]

Additional file 1:Table S1 Reads quality of miRNA libraries.

| Sample | Reads | Clean reads | Q20 | Q30 | GC content |
| --- | --- | --- | --- | --- | --- |
| AI1 | 14742066 | 14368844 | 98.53% | 96.75% | 49.11% |
| AI2 | 13399090 | 12995792 | 98.57% | 96.84% | 48.72% |
| AI3 | 14486182 | 14057386 | 98.51% | 96.71% | 48.92% |
| CI1 | 13980369 | 13613482 | 98.48% | 96.66% | 48.91% |
| CI2 | 14520722 | 14122253 | 98.53% | 96.75% | 48.82% |
| CI3 | 13396115 | 13025246 | 98.37% | 96.45% | 48.97% |
| Con1 | 14771696 | 14351239 | 98.41% | 96.47% | 49.08% |
| Con2 | 12672519 | 12344811 | 98.52% | 96.71% | 49.10% |
| Con3 | 15026160 | 14647321 | 98.56% | 96.78% | 48.79% |
