# Supplementary material for: miRNA and circRNA expression patterns in mouse brain during toxoplasmosis development
Source: BMC Genomics. 2020 Jan 14;21:46. doi: 10.1186/s12864-020-6464-9 (PMC6958735; doi:10.1186/s12864-020-6464-9)
Supplement: Supplementary file 2 — Additional file 2: Table S2. Statistics of small RNA reads mapped to the reference sequence. [file 12864_2020_6464_MOESM2_ESM.doc]

**Additional file 2: Table S2** Statistics of Small RNA reads mapped to the reference sequence.

| Sample | Total sRNA | Mapped sRNA | Mapped sRNA(+) | Mapped sRNA(-) |
| --- | --- | --- | --- | --- |
| AI1 | 13611279 | 12846575 | 9284785 | 3561790 |
| AI2 | 12764536 | 12012381 | 8937989 | 3074392 |
| AI3 | 12764536 | 12012381 | 8937989 | 3074392 |
| CI1 | 13179394 | 12372633 | 9048698 | 3323935 |
| CI2 | 13754977 | 12895612 | 10036077 | 3116677 |
| CI3 | 12610351 | 11765919 | 8759079 | 3006840 |
| Con1 | 13930639 | 13152754 | 10036077 | 3116677 |
| Con2 | 11973374 | 11301212 | 8820977 | 2480235 |
| Con3 | 14272818 | 13457367 | 10163028 | 3294339 |

The symbols in brackets indicate the counts of each mapped sRNA in the forward (+)and reverse (-) orientations.
