# Supplementary material for: miRNA and circRNA expression patterns in mouse brain during toxoplasmosis development
Source: BMC Genomics. 2020 Jan 14;21:46. doi: 10.1186/s12864-020-6464-9 (PMC6958735; doi:10.1186/s12864-020-6464-9)
Supplement: Supplementary file 3 — Additional file 3: Table S3. RNA reads counts in the infected and control brains. [file 12864_2020_6464_MOESM3_ESM.doc]

**Additional file 3: Table S****3** Small RNA reads counts in the infected and control brains.

| Types | AI1 | AI2 | AI3 | CI1 | CI2 | CI3 | Con1 | Con2 | Con3 |
| --- | --- | --- | --- | --- | --- | --- | --- | --- | --- |
| total | 12846575 | 12012381 | 12894572 | 12372633 | 12895612 | 11765919 | 13152754 | 11301212 | 13457367 |
| known_miRNA | 8744162 | 8287617 | 8915261 | 8166864 | 8561148 | 7700507 | 9098879 | 7799882 | 9341442 |
| rRNA | 143032 | 114999 | 152226 | 149517 | 165225 | 191198 | 112255 | 112495 | 136143 |
| tRNA | 11672 | 11724 | 11174 | 14649 | 11486 | 10341 | 9234 | 8297 | 12073 |
| snRNA | 3705 | 2039 | 2699 | 4293 | 3784 | 4196 | 2168 | 2099 | 2201 |
| snoRNA | 67838 | 83127 | 84200 | 84148 | 116024 | 86581 | 61237 | 49405 | 73130 |
| repeat | 83795 | 59801 | 70108 | 72685 | 88120 | 95006 | 68513 | 52153 | 67626 |
| novel_miRNA | 1419 | 1388 | 1443 | 1194 | 1397 | 1302 | 1389 | 1399 | 2113 |
| exon:+ | 141906 | 118662 | 153288 | 131105 | 166240 | 180663 | 116655 | 104355 | 116219 |
| exon:- | 5742 | 3364 | 3966 | 4586 | 4377 | 4463 | 5228 | 4141 | 4005 |
| intron:+ | 184049 | 97586 | 152539 | 160946 | 182412 | 220335 | 118012 | 125100 | 128086 |
| intron:- | 15192 | 14913 | 15635 | 16601 | 20847 | 20493 | 12209 | 10798 | 13962 |
| other | 3444063 | 3217161 | 3332033 | 3566045 | 3574552 | 3250834 | 3546975 | 3031088 | 3560367 |

‘intron:+’ or ‘intron:-’ represents sRNA sequence derived from sense or [antisense](https://www.sciencedirect.com/topics/medicine-and-dentistry/antisense) of [intron](https://www.sciencedirect.com/topics/biochemistry-genetics-and-molecular-biology/intron) respectively;’ exon:+’ or ‘exon:-’ represents small RNA derived from sense or antisense of [exon](https://www.sciencedirect.com/topics/biochemistry-genetics-and-molecular-biology/exon) respectively.
