# Supplementary figures and images for: miRNA and circRNA expression patterns in mouse brain during toxoplasmosis development
Source: BMC Genomics. 2020 Jan 14;21:46. doi: 10.1186/s12864-020-6464-9 (PMC6958735; doi:10.1186/s12864-020-6464-9)

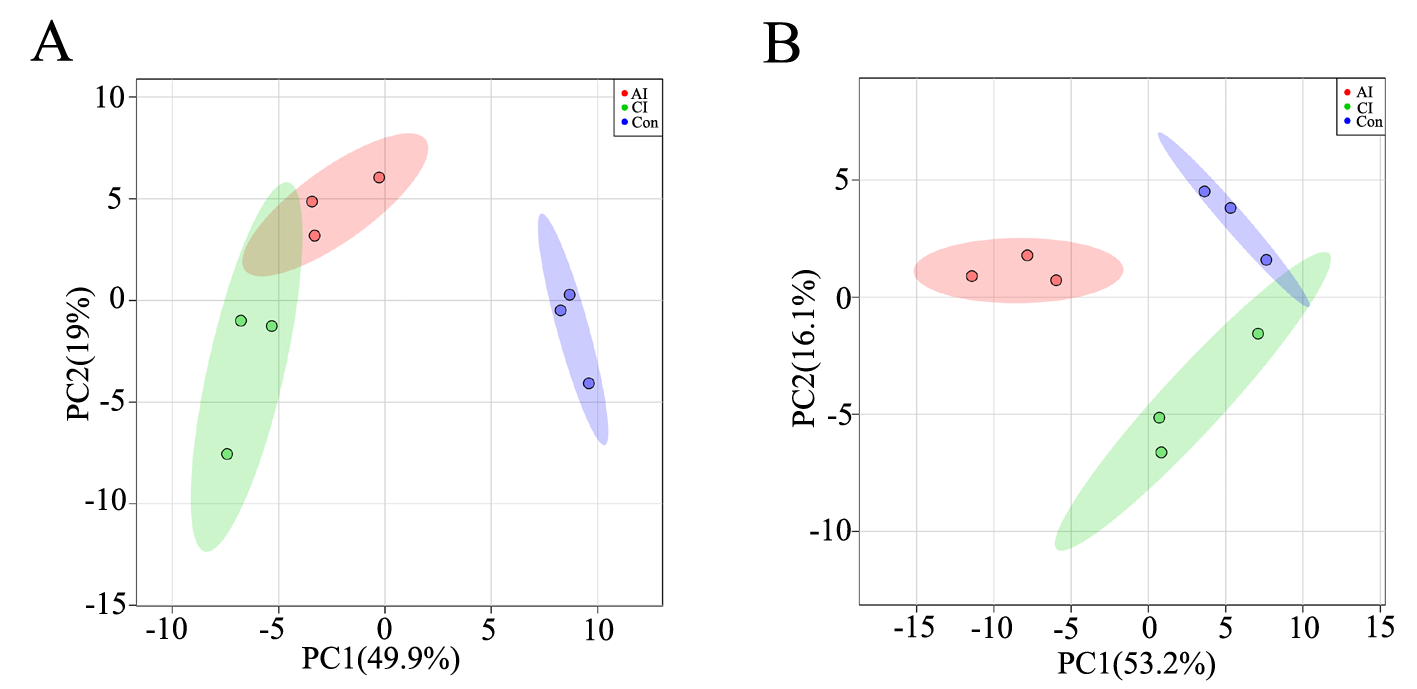

Supplement: Supplementary file 4 — Additional file 4: Figure S1. Principal Component Analysis (PCA) score scatter plots of miRNA (A) and circRNA (B) obtained from high-throughput RNA sequencing. Ellipses enclose the 95% confidence intervals estimated by the sample means and covariances of each group. [file 12864_2020_6464_MOESM4_ESM.tif]

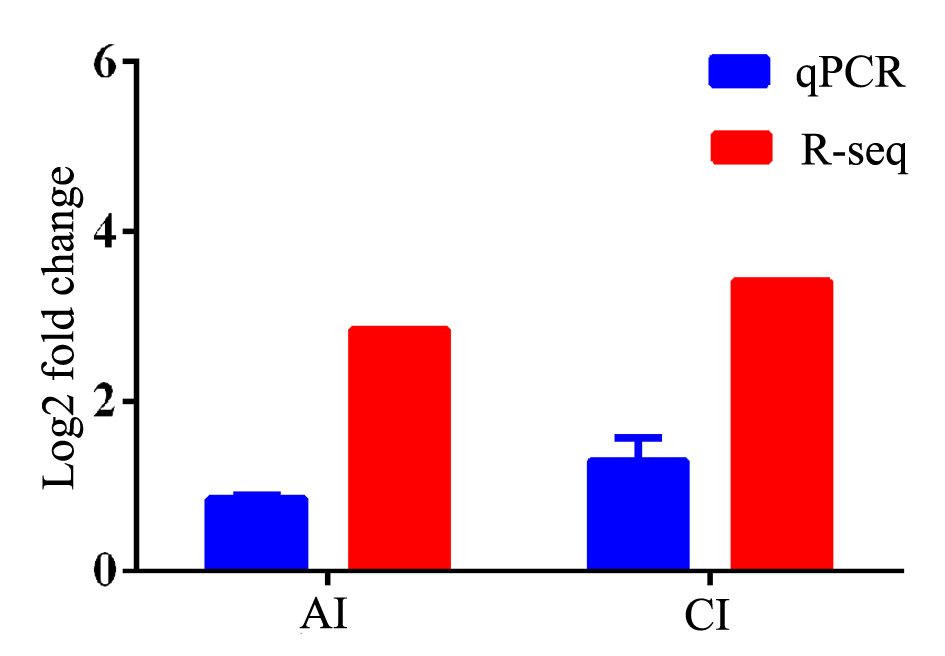

Supplement: Supplementary file 5 — Additional file 5: Figure S2. Validation of the expression of mmu-miR-155-5p using qPCR analysis. Y axis indicates the relative expression with log2 fold change. [file 12864_2020_6464_MOESM5_ESM.tif]
