# Supplementary material for: miRNA and circRNA expression patterns in mouse brain during toxoplasmosis development
Source: BMC Genomics. 2020 Jan 14;21:46. doi: 10.1186/s12864-020-6464-9 (PMC6958735; doi:10.1186/s12864-020-6464-9)
Supplement: Supplementary file 6 — Additional file 6: Table S4. Reads quality of circRNA libraries. [file 12864_2020_6464_MOESM6_ESM.doc]

Table S4 Reads quality of circRNA libraries.

| Sample name | Raw reads | Clean reads | clean bases | Error rate(%) | Q20(%) | Q30(%) | GC content(%) |
| --- | --- | --- | --- | --- | --- | --- | --- |
| AI1 | 68821568 | 66799066 | 10.02G | 0.02 | 98.32 | 95.54 | 60.45 |
| AI2 | 68311012 | 66461678 | 9.96G | 0.02 | 98.38 | 95.65 | 60.52 |
| AI3 | 66413072 | 64618174 | 9.70G | 0.02 | 98.28 | 95.47 | 60.51 |
| CI1 | 61862252 | 60457932 | 9.06G | 0.02 | 98.2 | 95.39 | 60.67 |
| CI2 | 55090200 | 53825936 | 8.08G | 0.02 | 98.19 | 95.37 | 60.95 |
| CI3 | 60452188 | 59065714 | 8.86G | 0.02 | 98.2 | 95.38 | 60.61 |
| Con1 | 54095406 | 53812656 | 8.08G | 0.03 | 96.56 | 91.31 | 59.91 |
| Con2 | 59831286 | 59501906 | 8.92G | 0.03 | 96.56 | 91.33 | 60.07 |
| Con3 | 62533400 | 62033874 | 9.30G | 0.03 | 96.78 | 92.22 | 60.75 |
