# Supplementary material for: miRNA and circRNA expression patterns in mouse brain during toxoplasmosis development
Source: BMC Genomics. 2020 Jan 14;21:46. doi: 10.1186/s12864-020-6464-9 (PMC6958735; doi:10.1186/s12864-020-6464-9)
Supplement: Supplementary file 9 — Additional file 9: Table S6. Differentially expressed circRNAs and their corresponding host genes. [file 12864_2020_6464_MOESM9_ESM.doc]

**Additional file 9: Table S6** Differentially expressed circRNAs and their corressponding host genes.

| Comparison groups | Differential circRNAs | Host genes |
| --- | --- | --- |
| Acute vs Control | novel_circ_0000059 | ENSMUSG00000036019 |
|  | novel_circ_0001656 | ENSMUSG00000096054 |
|  | novel_circ_0002091 | ENSMUSG00000015202 |
|  | novel_circ_0002092 | ENSMUSG00000015202 |
|  | novel_circ_0002626 | ENSMUSG00000058589 |
|  | novel_circ_0004884 | ENSMUSG00000055333 |
|  | novel_circ_0005150 | ENSMUSG00000018501 |
|  | novel_circ_0005368 | ENSMUSG00000020431 |
|  | novel_circ_0006107 | n/a |
|  | novel_circ_0008938 | ENSMUSG00000021065 |
|  | novel_circ_0009548 | ENSMUSG00000033854 |
|  | novel_circ_0009550 | ENSMUSG00000033854 |
|  | novel_circ_0009555 | ENSMUSG00000033854 |
|  | novel_circ_0012375 | ENSMUSG00000041650 |
|  | novel_circ_0014664 | ENSMUSG00000040420 |
|  | novel_circ_0015196 | ENSMUSG00000037386 |
|  | novel_circ_0015204 | ENSMUSG00000037386 |
|  | novel_circ_0015206 | ENSMUSG00000037386 |
|  | novel_circ_0015210 | ENSMUSG00000037386 |
|  | novel_circ_0018046 | ENSMUSG00000022687 |
|  | novel_circ_0018357 | ENSMUSG00000052504 |
|  | novel_circ_0018807 | ENSMUSG00000022946 |
|  | novel_circ_0019376 | ENSMUSG00000063239 |
|  | novel_circ_0019378 | ENSMUSG00000063239 |
|  | novel_circ_0019380 | ENSMUSG00000063239 |
|  | novel_circ_0019383 | ENSMUSG00000063239 |
|  | novel_circ_0019385 | ENSMUSG00000063239 |
|  | novel_circ_0019387 | ENSMUSG00000040276 |
|  | novel_circ_0020455 | ENSMUSG00000024066 |
|  | novel_circ_0024328 | ENSMUSG00000025083 |
|  | novel_circ_0024340 | ENSMUSG00000025085 |
|  | novel_circ_0027754 | ENSMUSG00000026098 |
|  | novel_circ_0027979 | ENSMUSG00000026024 |
|  | novel_circ_0029340 | ENSMUSG00000057378 |
|  | novel_circ_0032234 | ENSMUSG00000026915 |
|  | novel_circ_0034114 | ENSMUSG00000027965 |
|  | novel_circ_0034279 | ENSMUSG00000053965 |
|  | novel_circ_0034609 | ENSMUSG00000040253 |
|  | novel_circ_0036454 | ENSMUSG00000027508 |
|  | novel_circ_0036503 | ENSMUSG00000035305 |
|  | novel_circ_0038052 | n/a |
|  | novel_circ_0039389 | ENSMUSG00000028373 |
|  | novel_circ_0039396 | ENSMUSG00000028373 |
|  | novel_circ_0039606 | ENSMUSG00000041261 |
|  | novel_circ_0039796 | ENSMUSG00000041235 |
|  | novel_circ_0040467 | ENSMUSG00000063430 |
|  | novel_circ_0042094 | ENSMUSG00000039934 |
|  | novel_circ_0042934 | ENSMUSG00000085720 |
|  | novel_circ_0043131 | ENSMUSG00000029189 |
|  | novel_circ_0043135 | ENSMUSG00000029189 |
|  | novel_circ_0043845 | ENSMUSG00000060961 |
|  | novel_circ_0044835 | ENSMUSG00000030199 |
|  | novel_circ_0045342 | ENSMUSG00000017978 |
|  | novel_circ_0046296 | ENSMUSG00000004633 |
|  | novel_circ_0046297 | ENSMUSG00000004633 |
|  | novel_circ_0046339 | ENSMUSG00000004347 |
|  | novel_circ_0046345 | ENSMUSG00000004347 |
|  | novel_circ_0046488 | ENSMUSG00000071424 |
|  | novel_circ_0047285 | ENSMUSG00000030718 |
|  | novel_circ_0048152 | ENSMUSG00000030956 |
|  | novel_circ_0048158 | ENSMUSG00000030956 |
|  | novel_circ_0048179 | ENSMUSG00000030849 |
|  | novel_circ_0049270 | n/a |
|  | novel_circ_0050766 | ENSMUSG00000053399 |
|  | novel_circ_0051893 | ENSMUSG00000031608 |
|  | novel_circ_0051963 | ENSMUSG00000037921 |
|  | novel_circ_0052574 | ENSMUSG00000048617 |
|  | novel_circ_0052695 | ENSMUSG00000097248 |
|  | novel_circ_0054350 | ENSMUSG00000032036 |
|  | novel_circ_0054356 | ENSMUSG00000032036 |
|  | novel_circ_0055418 | ENSMUSG00000036466 |
|  | novel_circ_0055422 | ENSMUSG00000036466 |
|  | novel_circ_0055646 | ENSMUSG00000032376 |
|  | novel_circ_0055998 | ENSMUSG00000062151 |
|  | novel_circ_0057656 | ENSMUSG00000037341 |
|  | novel_circ_0057684 | ENSMUSG00000036769 |
| Chronic vs Control | novel_circ_0019981 | ENSMUSG00000004730 |
|  | novel_circ_0022887 | ENSMUSG00000024548 |
|  | novel_circ_0057684 | ENSMUSG00000036769 |
