# Supplementary material for: miRNA and circRNA expression patterns in mouse brain during toxoplasmosis development
Source: BMC Genomics. 2020 Jan 14;21:46. doi: 10.1186/s12864-020-6464-9 (PMC6958735; doi:10.1186/s12864-020-6464-9)
Supplement: Supplementary file 10 — Additional file 10: Table S7. The data used to construct Fig. 6. [file 12864_2020_6464_MOESM10_ESM.docx]

**Additional file 10: Table S7** The data used to construct Figure 6.

| miRNA | Log2 fold change | circRNA | Log2 fold change | Combined interaction score |
| --- | --- | --- | --- | --- |
| mmu-let-7b-5p | -0.24822 | novel_circ_0019376 | -1.0089 | 147 |
| mmu-let-7b-5p | -0.24822 | novel_circ_0019378 | -1.6645 | 147 |
| mmu-let-7b-5p | -0.24822 | novel_circ_0019380 | -1.6051 | 147 |
| mmu-let-7b-5p | -0.24822 | novel_circ_0042934 | -1.1018 | 154 |
| mmu-let-7c-5p | -0.28234 | novel_circ_0019376 | -1.0089 | 140 |
| mmu-let-7c-5p | -0.28234 | novel_circ_0019378 | -1.6645 | 140 |
| mmu-let-7c-5p | -0.28234 | novel_circ_0019380 | -1.6051 | 140 |
| mmu-let-7c-5p | -0.28234 | novel_circ_0042934 | -1.1018 | 154 |
| mmu-let-7d-5p | -0.33807 | novel_circ_0019376 | -1.0089 | 140 |
| mmu-let-7d-5p | -0.33807 | novel_circ_0019378 | -1.6645 | 140 |
| mmu-let-7d-5p | -0.33807 | novel_circ_0019380 | -1.6051 | 140 |
| mmu-let-7d-5p | -0.33807 | novel_circ_0042934 | -1.1018 | 147 |
| mmu-miR-10b-5p | -1.8485 | novel_circ_0006107 | -1.463 | 148 |
| mmu-miR-10b-5p | -1.8485 | novel_circ_0009550 | -0.96904 | 142 |
| mmu-miR-10b-5p | -1.8485 | novel_circ_0009555 | -1.0423 | 142 |
| mmu-miR-125a-5p | -0.38131 | novel_circ_0001656 | -1.2696 | 145 |
| mmu-miR-129-1-3p | -0.35333 | novel_circ_0009550 | -0.96904 | 140 |
| mmu-miR-129-1-3p | -0.35333 | novel_circ_0009555 | -1.0423 | 140 |
| mmu-miR-129-1-3p | -0.35333 | novel_circ_0034114 | -1.6774 | 142 |
| mmu-miR-129-1-3p | -0.35333 | novel_circ_0038052 | -4.7277 | 150 |
| mmu-miR-129-1-3p | -0.35333 | novel_circ_0039396 | -0.91981 | 140 |
| mmu-miR-142a-3p | 2.0617 | novel_circ_0000059 | -1.1803 | 140 |
| mmu-miR-142b | 2.0617 | novel_circ_0015210 | -0.93818 | 146 |
| mmu-miR-146a-5p | 1.3578 | novel_circ_0020455 | 1.6428 | 140 |
| mmu-miR-146a-5p | 1.3578 | novel_circ_0050766 | -1.8664 | 145 |
| mmu-miR-147-3p | 1.4666 | novel_circ_0048152 | -2.9936 | 143 |
| mmu-miR-150-5p | -0.41416 | novel_circ_0006107 | -1.463 | 145 |
| mmu-miR-150-5p | -0.41416 | novel_circ_0036454 | -1.3659 | 145 |
| mmu-miR-155-5p | 2.8493 | novel_circ_0008938 | -1.0248 | 151 |
| mmu-miR-17-5p | 0.42387 | novel_circ_0018357 | -1.2729 | 167 |
| mmu-miR-17-5p | 0.42387 | novel_circ_0038052 | -4.7277 | 171 |
| mmu-miR-17-5p | 0.42387 | novel_circ_0046296 | -1.335 | 160 |
| mmu-miR-17-5p | 0.42387 | novel_circ_0046297 | -1.9884 | 160 |
| mmu-miR-181a-5p | -0.31414 | novel_circ_0027754 | -0.50295 | 148 |
| mmu-miR-181a-5p | -0.31414 | novel_circ_0032234 | 0.57879 | 162 |
| mmu-miR-181a-5p | -0.31414 | novel_circ_0036503 | -1.8209 | 152 |
| mmu-miR-195a-5p | -0.88926 | novel_circ_0009548 | -1.1825 | 141 |
| mmu-miR-195a-5p | -0.88926 | novel_circ_0009550 | -0.96904 | 141 |
| mmu-miR-195a-5p | -0.88926 | novel_circ_0039796 | -0.91981 | 149 |
| mmu-miR-195a-5p | -0.88926 | novel_circ_0042094 | 1.3702 | 162 |
| mmu-miR-195a-5p | -0.88926 | novel_circ_0046339 | -1.3354 | 143 |
| mmu-miR-195a-5p | -0.88926 | novel_circ_0049270 | 2.143 | 143 |
| mmu-miR-1a-3p | -0.60676 | novel_circ_0047285 | 1.2793 | 142 |
| mmu-miR-1a-3p | -0.60676 | novel_circ_0048152 | -2.9936 | 140 |
| mmu-miR-1b-5p | -0.60719 | novel_circ_0027754 | -0.50295 | 156 |
| mmu-miR-1b-5p | -0.60719 | novel_circ_0034279 | -0.96563 | 148 |
| mmu-miR-203-3p | 1.5746 | novel_circ_0015196 | -1.2222 | 151 |
| mmu-miR-203-3p | 1.5746 | novel_circ_0057684 | -1.9747 | 157 |
| mmu-miR-206-3p | -2.2613 | novel_circ_0047285 | 1.2793 | 142 |
| mmu-miR-206-3p | -2.2613 | novel_circ_0048152 | -2.9936 | 155 |
| mmu-miR-20a-5p | 0.5352 | novel_circ_0018357 | -1.2729 | 163 |
| mmu-miR-20a-5p | 0.5352 | novel_circ_0038052 | -4.7277 | 171 |
| mmu-miR-20a-5p | 0.5352 | novel_circ_0046296 | -1.335 | 156 |
| mmu-miR-20a-5p | 0.5352 | novel_circ_0046297 | -1.9884 | 156 |
| mmu-miR-20b-5p | 1.1462 | novel_circ_0018357 | -1.2729 | 159 |
| mmu-miR-20b-5p | 1.1462 | novel_circ_0038052 | -4.7277 | 175 |
| mmu-miR-20b-5p | 1.1462 | novel_circ_0046296 | -1.335 | 155 |
| mmu-miR-20b-5p | 1.1462 | novel_circ_0046297 | -1.9884 | 155 |
| mmu-miR-214-3p | -0.94522 | novel_circ_0001656 | -1.2696 | 310 |
| mmu-miR-214-3p | -0.94522 | novel_circ_0006107 | -1.463 | 154 |
| mmu-miR-214-3p | -0.94522 | novel_circ_0018046 | -0.91577 | 145 |
| mmu-miR-214-3p | -0.94522 | novel_circ_0019376 | -1.0089 | 140 |
| mmu-miR-214-3p | -0.94522 | novel_circ_0019378 | -1.6645 | 140 |
| mmu-miR-214-3p | -0.94522 | novel_circ_0019380 | -1.6051 | 140 |
| mmu-miR-214-3p | -0.94522 | novel_circ_0027979 | -1.1815 | 161 |
| mmu-miR-214-3p | -0.94522 | novel_circ_0029340 | 0.63253 | 158 |
| mmu-miR-214-3p | -0.94522 | novel_circ_0040467 | -0.52929 | 152 |
| mmu-miR-214-3p | -0.94522 | novel_circ_0054356 | -1.3737 | 140 |
| mmu-miR-214-3p | -0.94522 | novel_circ_0055646 | -0.87953 | 150 |
| mmu-miR-219a-2-3p | -0.54216 | novel_circ_0034279 | -0.96563 | 142 |
| mmu-miR-21a-3p | 1.1803 | novel_circ_0009548 | -1.1825 | 156 |
| mmu-miR-21a-3p | 1.1803 | novel_circ_0009550 | -0.96904 | 156 |
| mmu-miR-21a-3p | 1.1803 | novel_circ_0018357 | -1.2729 | 163 |
| mmu-miR-21a-3p | 1.1803 | novel_circ_0027979 | -1.1815 | 151 |
| mmu-miR-21a-3p | 1.1803 | novel_circ_0044835 | 1.1373 | 146 |
| mmu-miR-21a-3p | 1.1803 | novel_circ_0048152 | -2.9936 | 140 |
| mmu-miR-21a-3p | 1.1803 | novel_circ_0055646 | -0.87953 | 149 |
| mmu-miR-223-3p | 2.1068 | novel_circ_0057684 | -1.9747 | 162 |
| mmu-miR-223-5p | 2.2766 | novel_circ_0038052 | -4.7277 | 154 |
| mmu-miR-27a-3p | 0.31296 | novel_circ_0014664 | -1.1756 | 140 |
| mmu-miR-27a-3p | 0.31296 | novel_circ_0036503 | -1.8209 | 303 |
| mmu-miR-27a-3p | 0.31296 | novel_circ_0039396 | -0.91981 | 156 |
| mmu-miR-3065-3p | -0.48383 | novel_circ_0001656 | -1.2696 | 158 |
| mmu-miR-3065-3p | -0.48383 | novel_circ_0006107 | -1.463 | 175 |
| mmu-miR-3065-3p | -0.48383 | novel_circ_0019387 | -0.63494 | 143 |
| mmu-miR-3065-3p | -0.48383 | novel_circ_0055422 | -1.3685 | 146 |
| mmu-miR-34a-5p | -1.0237 | novel_circ_0020455 | 1.6428 | 142 |
| mmu-miR-34a-5p | -1.0237 | novel_circ_0027754 | -0.50295 | 289 |
| mmu-miR-34a-5p | -1.0237 | novel_circ_0047285 | 1.2793 | 152 |
| mmu-miR-34a-5p | -1.0237 | novel_circ_0048152 | -2.9936 | 152 |
| mmu-miR-363-3p | 1.0312 | novel_circ_0045342 | -1.3004 | 143 |
| mmu-miR-363-3p | 1.0312 | novel_circ_0054356 | -1.3737 | 152 |
| mmu-miR-455-3p | -0.77907 | novel_circ_0002091 | -1.3953 | 144 |
| mmu-miR-455-3p | -0.77907 | novel_circ_0002092 | -1.6499 | 144 |
| mmu-miR-455-3p | -0.77907 | novel_circ_0008938 | -1.0248 | 150 |
| mmu-miR-455-3p | -0.77907 | novel_circ_0014664 | -1.1756 | 144 |
| mmu-miR-455-3p | -0.77907 | novel_circ_0032234 | 0.57879 | 147 |
| mmu-miR-455-3p | -0.77907 | novel_circ_0043131 | -0.74982 | 142 |
| mmu-miR-455-3p | -0.77907 | novel_circ_0044835 | 1.1373 | 154 |
| mmu-miR-484 | -0.60984 | novel_circ_0024328 | -1.4065 | 141 |
| mmu-miR-484 | -0.60984 | novel_circ_0040467 | -0.52929 | 143 |
| mmu-miR-484 | -0.60984 | novel_circ_0048152 | -2.9936 | 176 |
| mmu-miR-484 | -0.60984 | novel_circ_0055422 | -1.3685 | 148 |
| mmu-miR-497a-5p | -0.58161 | novel_circ_0009548 | -1.1825 | 162 |
| mmu-miR-497a-5p | -0.58161 | novel_circ_0009550 | -0.96904 | 162 |
| mmu-miR-497a-5p | -0.58161 | novel_circ_0039796 | -0.91981 | 151 |
| mmu-miR-497a-5p | -0.58161 | novel_circ_0042094 | 1.3702 | 144 |
| mmu-miR-497a-5p | -0.58161 | novel_circ_0046339 | -1.3354 | 151 |
| mmu-miR-497a-5p | -0.58161 | novel_circ_0049270 | 2.143 | 154 |
| mmu-miR-5114 | 1.1698 | novel_circ_0032234 | 0.57879 | 144 |
| mmu-miR-5114 | 1.1698 | novel_circ_0034114 | -1.6774 | 140 |
| mmu-miR-5114 | 1.1698 | novel_circ_0034609 | 1.4652 | 140 |
| mmu-miR-5114 | 1.1698 | novel_circ_0038052 | -4.7277 | 152 |
| mmu-miR-5114 | 1.1698 | novel_circ_0051893 | -1.3688 | 323 |
| mmu-miR-5114 | 1.1698 | novel_circ_0055646 | -0.87953 | 153 |
| mmu-miR-598-3p | -0.29674 | novel_circ_0048152 | -2.9936 | 155 |
| mmu-miR-615-3p | -1.5411 | novel_circ_0024328 | -1.4065 | 149 |
| mmu-miR-615-3p | -1.5411 | novel_circ_0051893 | -1.3688 | 145 |
| mmu-miR-744-5p | -0.39267 | novel_circ_0019387 | -0.63494 | 158 |
| mmu-miR-744-5p | -0.39267 | novel_circ_0040467 | -0.52929 | 153 |
| mmu-miR-744-5p | -0.39267 | novel_circ_0055422 | -1.3685 | 159 |
| mmu-miR-881-3p | -0.93269 | novel_circ_0034114 | -1.6774 | 142 |
| mmu-miR-881-3p | -0.93269 | novel_circ_0042094 | 1.3702 | 163 |
| mmu-miR-881-3p | -0.93269 | novel_circ_0055646 | -0.87953 | 160 |
