# Supplementary material for: miRNA and circRNA expression patterns in mouse brain during toxoplasmosis development
Source: BMC Genomics. 2020 Jan 14;21:46. doi: 10.1186/s12864-020-6464-9 (PMC6958735; doi:10.1186/s12864-020-6464-9)
Supplement: Supplementary file 13 — Additional file 13: Table S8. Primers for miRNA and circRNA q-PCR analysis. [file 12864_2020_6464_MOESM13_ESM.doc]

**Additional file 13: Table S8** Primers for miRNA and circRNA q-PCR analysis.

| Primers | Sequence(5’-3’) |
| --- | --- |
| mmu-miR-155-5p-F | CCGCGTTAATGCTAATTGTGATAGGGGT |
| mmu-miR-21a-3p-F | caacagcagtcgatgggctgtc |
| mmu-miR-455-3p-F | gcagtccacgggcatatacac |
| mmu-miR-497a-5p-F | cagcagcacactgtggtttgta |
| mmu-miR-214-3p-F | acagcaggcacagacaggcagt |
| U6-F | GGAACGATACAGAGAAGATTAGC |
| U6-R | TGGAACGCTTCACGAATTTGCG |
| novel_circ_0019383-F | GCCTGGTATGTGTCCGAGG |
| novel_circ_0019383-R | AGAACGGTGAGTGGTGTGTG |
| novel_circ_0046297-F | AAGGAAAGCTAGCGTCAGCA |
| novel_circ_0046297-R | GGATGTAGGCACCTTCCACG |
| novel_circ_0044835-F | TGAGTCCACAGCTTCAGATGG |
| novel_circ_0044835-R | TATGGAGGCGCTTCTCTGTTG |
| novel_circ_0029340-F | TGTGCTTTGTAGGTGACCCAA |
| novel_circ_0029340-R | CGGAACACAACCGCCTTATTC |
| novel_circ_0048152-184-F | GGAGCCATCCTGACACTTTTC |
| novel_circ_0048152-184-R | AGAAGATCGGTGTGAAACGGC |
| GAPDH-F | GGACACTGAGCAAGAGAGGC |
| GAPDH-R | TTATGGGGGTCTGGGATGGA |
| Mus-Serping1-F | GAACTTGGACCAGGACGCAG |
| Mus-Serping1-R | GAGAAGGCTCTATCCCCAGC |
| Mus-Trim21-F | CCTGGTTAGATTCCACGGCA |
| Mus-Trim21-R | GCCCCCATTCTTCCCAACTT |
| Mus-Dgcr6-F | CCCCTTGACTGGATGCTGAG |
| Mus-Dgcr6-R | TTCTAGGCCATCCTCTGGCT |
| Mus-Dgkz-F | ATCCCAGCAAACTGGAGCAA |
| Mus-Dgkz-R | GGGCTGATCTGGAAGGACAA |
| Mus-Irf1-F | GCAAACTTCCGTTGTGCCAT |
| Mus-Irf1-R | TCGGCTGGACTTGGACTTTC |
| Mus-Ifi44-F | CCCATGACCCACTGCTGAA |
| Mus-Ifi44-R | TAAAGCCAAATGCAGAATGCCA |
| Mus-Tgfbr1-F | AGTCAGTCCGTTGGGTCTTC |
| Mus-Tgfbr1-R | TAACATTTCCCCGCCCAGAAT |
